# Physicians’ and Nurses’ Perceptions of Pharmacists’ Competencies, and Their Needs of Pharmacists during COVID-19

**DOI:** 10.3390/pharmacy10030064

**Published:** 2022-06-13

**Authors:** Kayoko Takeda Mamiya, Kazumasa Hirata

**Affiliations:** 1 Faculty of Pharmaceutical Sciences, Hokkaido University of Science, Sapporo 006-8585, Japan; 2 School of Pharmaceutical Sciences, Wakayama Medical University, Wakayama 640-8156, Japan; khirata@wakayama-med.ac.jp

**Keywords:** perception study, pharmacists, physician, nurse, assessment, COVID-19

## Abstract

Objective: To understand how physicians and nurses evaluate Japanese pharmacists’ observed competencies and to explore potential new roles for pharmacists during COVID-19. Methods: A web-based Japanese survey with 25 items assessing physicians’ and nurses’ workplaces and the degree of their relationship with pharmacists in their daily work was conducted (Intage, Inc., Tokyo, Japan) in Japan in June 2021 (for one week beginning on 22 June). The survey asked physicians and nurses whether pharmacists had the required professional competencies and whether the needs of physicians and nurses were met by pharmacists in their workplaces. The scored questionnaire data, which used a Likert scale, were calculated as the mean and standard deviation (S.D.). The perception assessment scale used four levels (1, Agree; 2, Slightly agree; 3, Slightly disagree; and 4, Disagree). Results: This perception study ultimately obtained responses from 304 physicians and 336 nurses. Most pharmacists’ competencies were evaluated as “Agree” or “Slightly agree” by the physicians and nurses. However, the competencies for “Fundamental basic science” and “Prescription analytical skill or case analytical skill” were evaluated significantly lower by physicians than by nurses (Mann–Whitney U test, *p* < 0.01). Regarding physicians’ and nurses’ needs from pharmacists, nurses hoped that pharmacists could play a greater role as healthcare professionals in response to all items; in contrast, physicians hoped that pharmacists could play a greater role as healthcare professionals in response to five items. The common items were related to the role of healthcare professionals in the community. Conclusion: Our research is necessary for facilitating interprofessional collaboration and reflecting these results in pharmacy education by allowing physicians and nurses to assess the competencies of pharmacists and to understand their needs; however, these data are from only one country.

## 1. Introduction

During the spread of COVID-19, both citizens and healthcare professionals became more interested in infection prevention and public health, and the role of Japanese pharmacists began gradually changing. Japanese pharmacists are currently unable to vaccinate, but discussions have begun on the possibility of pharmacists playing such a role in the future [1]. A previous study of Japanese citizens during COVID-19 indicated that many citizens had not received education/training on infection prevention (hand washing or disinfection), and 89.7% (269/300 respondents) of citizens stated that their “infection prevention awareness increased” after the spread of COVID-19 [2]. In addition, citizens’ perceptions of infection prevention were shown to increase, with 47.7% (143/300 respondents) of citizens wanting to receive education/training on the methods and effects of infection prevention from healthcare professionals. However, of these, only 21.7% wanted to receive education/training from pharmacists [2]. This low rate (21.7%) could be due to a lack of public awareness of the role that pharmacists play in public health, especially in infection prevention, as pharmacists have not sufficiently played a role in public health in the community until now. However, if the perception and activities of community pharmacists change in the future, the perception of Japanese citizens may also change. Such changes in the role of pharmacists are occurring not only in Japan but also in the USA and China [3,4].

The Japanese School Education Act was revised in 2004, and pharmacy education was changed from a four- to a six-year program in 2006 [5]. This substantial reform indicated that becoming a pharmacist requires not only sufficient practical training but also specialization and a demonstrated sense of commitment to contributing to patient care. However, after this model core curriculum was implemented, several problems emerged (there are many items in this model core curriculum, making it difficult for each university to set out its own uniqueness, etc.); thus, the model core curriculum was revised with an approximate 25% reduction in the amount of content. The revised model core curriculum was implemented in 2015 [6]. However, another updated model core curriculum will be implemented in Japan in 2024. This curriculum is currently undergoing revision to meet the needs of society and regulators, especially with respect to the role of public health in the community.

The role of pharmacists is changing as social needs change, and education must meet these needs [7]. Japan has a national health insurance system; almost all patients receive insurance prescriptions from physicians in hospitals, and all patients go to insurance pharmacies (except for hospitalized patients). In Japan, where the System of Separation of Dispensing and Prescribing Drugs has been underway since 1974, the percentage of the country covered by this system was 26.0% in 1997, 51.6% in 2003, and 74.9% in 2020 [8]. In the System of Separation of Dispensing and Prescribing Drugs, pharmacists must provide pharmacological management (including pharmacotherapy) to patients independently of the prescribing physician (including hospital pharmacists) [9]. This does not mean that only pharmacists are involved in pharmacotherapy but that pharmacists are the reviewers and last stop for pharmacotherapy. For this reason, insurance pharmacies and insurance medical institutions (except for the pharmacy departments of hospital facilities) have been prohibited from having an integrated structure as well as integrated management and must not be in a form where patients come and go by dedicated pathways, without public roads, etc. Since 2016, however, structural restrictions have been relaxed slightly due to various issues, such as wheelchair accessibility [10]. For the above reasons, pharmacists play an important role in healthcare, while they work less directly with multiple professions than nurses and other healthcare professionals and thus are less likely to receive direct feedback. Therefore, the assessment of the perceptions of physicians or nurses who work with pharmacists is very important for multidisciplinary collaboration, but we have not yet conducted a survey to investigate this issue in Japan. Moreover, few assessment surveys of physicians’ and nurses’ perceptions of pharmacist competency have been conducted worldwide, and many assessments are made by peer evaluations of pharmacists (experienced pharmacists evaluating new pharmacists) or pharmacy universities [11,12,13]. Therefore, in this study, we conducted a survey of physicians and nurses to determine whether pharmacists have the necessary professional competencies to collaborate with other healthcare professionals in the community. Moreover, we also asked the nurses and physicians what needs they had that could be fulfilled by pharmacists in their workplaces.

## 2. Materials and Methods

### 2.1. Study Design

A web-based Japanese survey with 25 items assessing physicians’ and nurses’ workplace and the degree of their relationship with pharmacists in their daily work was conducted (Intage, Inc., Tokyo, Japan) in Japan in June 2021 (for one week beginning on 22 June). We clearly stipulated the purpose of this research on the survey website. As of November 2020, this web research company that was utilized had 1413 physicians and 8376 nurses registered with their license number nationwide who were paid after completing a survey. Of the questionnaire items that were used, 10 concerned competencies required of a pharmacist at the time of graduation from undergraduate education based on the professional competencies for pharmacists as described in the basic policy of the revised 6-year model core curriculum [6]; we previously used these items in a pharmacist survey [14,15]. The other 12 questionnaire items were created based on the items related to the primary work of pharmacists. All questionnaire items are shown in Table 1 and Table 2 (physicians’ and nurses’ workplace and the degree of their relationship with pharmacists). The survey was administered only to respondents who understood and consented to the purpose of the present study, and no other exclusion criteria were established. This was a web-based survey, and we could not capture the rejection of consent; thus, we cannot calculate a valid response rate.

### 2.2. Ethical Approval

The ethics committee determined that it was not necessary to discuss ethical review in this study (application number: No21–15, July 2021). To protect personal information, this study was anonymous, and the data processed from the web survey did not contain any personal information; therefore, individuals could not be identified. Additionally, a code was established for the data file so that only study personnel could access the data via a specific personal computer.

### 2.3. Data Analysis

The scored questionnaire data, which used a Likert scale, were calculated as the mean and standard deviation (S.D.). The assessment scale used four levels (1, Agree; 2, Slightly agree; 3, Slightly disagree; and 4, Disagree). The Kolmogorov–Smirnov test was used to determine whether the data were normally distributed, and Mann–Whitney U tests were used to compare the assessments of physician respondents to those of nurse respondents. Principal component analysis was also used to confirm what the main requirements for pharmacists’ competencies were based on the responses of physicians and nurses. Communality was set to 1, and a principal component loading of 0.50 was used as the cut-off point. These analyses were performed using SPSS Statistics 25.0 (IBM, Chicago, IL, USA). Our research paper adheres to the ICMJE definition of authorship, and this article was written based on the SQUIRE 2.0 (Standards for Quality Improvement Reporting Excellence) guideline, which is considered to be the most suitable for our research.

## 3. Results

### 3.1. Demographics

The demographics of the respondents are shown in Table 2. We ultimately obtained answers from 304 physicians and 336 nurses. Their workplaces were clinics (physicians: 33.2%, *n* = 101; nurses: 32.4%, *n* = 109), university hospitals (physicians: 28.3%, *n* = 86; nurses: 29.8%, *n* = 100), national public hospitals (physicians: 21.7%, *n* = 66; nurses: 17.6%, *n* = 59) and general hospitals (physicians: 16.8%, *n* = 51; nurses: 20.2%, *n* = 68). Regarding their relationship with pharmacists in their daily work, more than 50% of physicians and nurses “sometimes” or “rarely” interacted with pharmacists.

### 3.2. Physicians’ and Nurses’ Perceptions of Pharmacists’ Competencies

We investigated the evaluations of pharmacists’ competencies, which were drawn from the responses of the assessment group of physicians and nurses. The results are shown in Table 3. Most competencies were evaluated as “Agree” or “Slightly agree” by the physicians or nurses. Most items had a mode of “Slightly agree”. Items on the characteristics of the pharmacist profession, including “Competency 5–2: Fundamental basic science” and “Competency 6–1: Prescription analytical skill or case analytical skill” were evaluated significantly lower by physicians than by nurses (Mann–Whitney U test, *p* < 0.01, Table 3). “Competency 5–2: Pharmaceutical knowledge” and “Competency 8: Research competency” were also evaluated significantly lower by physicians than by nurses, and only “Competency 2: Patient oriented attitude” was evaluated significantly higher by physicians than by nurses (Mann–Whitney U test, *p* < 0.01, Table 3).

### 3.3. Principal Component Analysis

To understand the competencies required by physicians and nurses of pharmacists, we conducted principal component analysis using the professional competencies for pharmacists defined by the Ministry of Education, Culture, Sports, Science and Technology (MEXT) (Questionnaire Items 1 to 10). For physicians, all items related to professional competency were categorized into two group domains that explained 63.6% of the cumulative contribution rate. Similarly, for nurses, all items related to professional competency were categorized into the same two group domains that explained 66.5% of the cumulative contribution rate. The two domains were named “component 1: professional competencies for pharmacists” (all 12 items) and “component 2: characteristic of pharmacists as specialists (two items selected by physicians and two items selected by nurses)”. These two domains, component 2, included the same items, “Competency 6–1: Prescription analytical skill or case analytical skill” and “Competency 5–1: More fundamental basic science”, for both physicians and nurses. A principal component loading of 0.50 was used as a cut-off point, and the results of the analysis ranged from 0.519 to 0.868. The results of the factor analysis with principal component analysis are presented in Table 4 and Table 5.

### 3.4. Required Role of Pharmacists in the Medical Field

To determine the pharmacist demands of physicians and nurses, we asked them whether there were more current needs in the medical field for which pharmacists could play a role. These results are shown in Table 6. Over 50% of nurses identified all items as areas in the medical field where pharmacists could be more involved (selecting either “Should work harder and play a greater role” or “Should play a greater role”), and similarly, over 50% of physicians chose the same specific areas where pharmacists could play a greater role: “genetic diagnosis/therapeutic drug monitoring (TDM)”, “disease prevention/public health in the community”, “pharmaceutical awareness activities for citizens”, “basic/clinical research for the development of medical care”, “medical contribution in drug discovery/as a scientist” and “drug management and patient monitoring related to clinical trials”. Overall, nurses tended to have stronger demands for pharmacists than physicians (Mann–Whitney U test, *p* < 0.01, Table 6).

## 4. Discussion

The role of healthcare professionals is changing with social needs and social changes. Therefore, both pharmacists and other healthcare professionals must adjust their roles to address their current needs. Healthcare professionals are required to change their roles after major behavior changes, such as those prompted by COVID-19. This study suggested that physicians and nurses would like pharmacists to take on additional roles in the community as needed.

In the perception assessment of physicians, over 60% of physicians assessed “Competency 5–1: Fundamental basic science” as low among pharmacists (slightly disagree or disagree). However, in these questionnaire items, we asked physicians whether pharmacists have a higher “fundamental basic science competency” than physicians. Therefore, it may be that physicians consider pharmacists to have a lower “fundamental basic science competency” than physicians even though they do possess this competency. On the other hand, in the 1990s and 2000s, according to the shift in the policy of the Ministry of Education, Culture, Sports, Science and Technology (MEXT) from ex ante regulation to ex post checks, the standards for the Establishment of Universities in Japan were revised, abolishing detailed statements about academic programs and relaxing the fundamental criteria. Subsequently, the number of pharmacy or pharmaceutical science schools increased from 46 in 2002 to 74 in 2016 [5,6,16]. The Japanese School Education Act was revised in 2004, and pharmacy education changed from a four- to a six-year program in 2006 [5]. This substantial reform indicated that becoming a pharmacist requires not only sufficient practical training but also specialization and a demonstrated sense of commitment to contributing to patient care. However, the deregulation allowed students who could not previously enter pharmacy universities to do so, and the extension of the educational year was intended to enrich practical experience in the six-year pharmacy education; however, this may also not have been enough to develop “fundamental basic science competency”. This means that the academic competencies of students have declined [17], which might be a legitimate assessment by physicians and a serious issue. Moreover, according to the principal component analysis results, it might be an expectation of physicians that pharmacists now play a greater role than before in “fundamental basic science competency” as science professionals. Whatever the reason, pharmacists need to improve “Competency 5–1: Fundamental basic science competency” more than ever in undergraduate education.

For “Competency 6–1: Prescription analytical skill or case analytical skill”, these competencies will be required to correct pharmacotherapy or pharmacological management, and pharmacists will collaborate with healthcare professionals through pharmacotherapy. At the same time, pharmacists will adapt their general pharmacotherapy knowledge for personalized pharmacotherapy through collaboration with other healthcare professionals. Therefore, most pharmacists might be surprised by this result. Indeed, in a previous study, experienced pharmacists assessed pharmacists who were educated in the new 6-year program as having “Competency 6–1: Prescription analytical skill or case analytical skill” [14]. The self-assessment by pharmacists who were educated by the new 6-year program also yielded the same result; they felt that they possessed “Competency 6–1: Prescription analytical skill or case analytical skill” [15]. However, the results of this study might suggest that interprofessional collaboration education is insufficient in Japanese pharmacy education. In a previous study on the same topics, experienced pharmacists or pharmacists who were educated by a new 6-year pharmacy education program evaluated “Competency 4: Interprofessional collaboration” as low [14,15]. This means that pharmacists may not be able to adapt personalized pharmacotherapy because they have insufficient experience in interprofessional collaboration in the medical field, including practical training in undergraduate pharmacy education, even though they learn general pharmacotherapy in undergraduate pharmacy education. Notably, interprofessional education is already being conducted in many countries [18,19]. The American Association of Colleges of Pharmacy indicates the core competencies for interprofessional collaborative practice [20], and interprofessional team-based learning is also incorporated into graduate medical education [19]. Moreover, in other countries, studies have shown that effective collaboration has been perceived to be beneficial to enhancing confidence in engagement and communication and increasing appreciation and respect for the expertise of other healthcare professionals; in contrast, critical patient information can be lost or misinterpreted if there is poor interprofessional communication and collaboration, contributing to poor patient outcomes [18]. Notably, some Japanese faculty of pharmacy have implemented interprofessional collaboration education with faculty of medicine; however, this applies to only a few faculties of pharmacy [21,22]. Therefore, we must consider both pre- and postgraduate education, which will develop “interprofessional collaboration education” in all pharmacy universities.

In the perception assessment of nurses, we found that they hoped that pharmacists could play a greater role as healthcare professionals in response to all items; in contrast, physicians hoped that pharmacists could play a greater role as healthcare professionals in response to five items (Table 6). These common items were related to the role of healthcare professionals in the community. In particular, “community disease prevention/public health” and “pharmaceutical awareness activities for citizens”, such as raising awareness about infection prevention and providing accurate information about vaccines, may not be sufficiently carried out by pharmacists at present during COVID-19. Along these lines, in a study in 2021, more than 70% (212/300) of pharmacists answered that they were not confident that they could correctly or appropriately instruct citizens in relation to “infection prevention methods or various vaccine information, etc.” [23]. Nevertheless, these activities are characteristic of their roles as pharmacists in the community. Indeed, in the Japanese medical system, most healthcare professionals involved in preillness/prevention are pharmacists. Physicians and nurses are involved when an individual becomes sick and goes to the hospital, but in other cases, the local pharmacy will address the individual’s needs. It should be noted that physicians and nurses might not understand pharmacist activities in the community; thus, information on pharmacists’ activities must be consciously provided through the Regional Comprehensive Support Center or the community to other healthcare professionals more than they are currently. In the USA, community pharmacists are knowledgeable and capable providers of public health services and are accessible and well regarded by the public [24]. Moreover, a previous Japanese study showed that 70% of citizen respondents (*n* = 300) had no education on infection prevention, and they hoped that citizens would want to receive infection prevention education from healthcare professionals [2]. Therefore, even considering the differences in the roles and rights of pharmacists by country, to sufficiently address the needs of physicians and nurses, Japanese pharmacists should consider increasing their contribution to public health in the community, especially in the area of infection prevention.

The gap in the evaluation of physicians and nurses might be due to differences in physicians’ or nurses’ roles in the community and in the medical field or the depth of involvement with pharmacists. In particular, one of the medical issues is the shortage of physicians or the uneven distribution of physicians in regions in Japan [25], and nurses are always the main support physicians in the medical field. In the System of Separation of Dispensing and Prescribing Drugs, although pharmacists work less directly with multiple professions than nurses and other healthcare professionals, pharmacists frequently have contact with physicians through nurses. Therefore, pharmacists are the most closely related to nurses in healthcare professionals. Our study results may suggest that nurses require the pharmacist role more than now, as nurses have already recognized pharmacists’ competencies.

Whatever the reasons, it may be necessary to clarify the perceptions gap between physicians and nurses or between healthcare professionals (physicians and nurses) and pharmacists through qualitative study. At the same time, we must consider the improvement of these competencies, “fundamental basic science”, “interprofessional collaboration”, and “contribution to public health in the community, especially, infection prevention” in new model core curriculum in 2024.

The limitation of our research is that we did not perform a qualitative study with physicians and nurses, and it is possible that we did not obtain enough data to fully assess their perceptions. However, in Japan, where the separation of medical and dispensary practice is being promoted, closely related tasks should be recognized as mutual roles, and the results of a small-scale qualitative study in Japan (a closed study that was not open to the public) of physicians who were working closely with pharmacists showed results similar to those of the present study. Therefore, in the future, we must clarify whether a qualitative study in Japan of physicians or nurses who work closely with pharmacists would show results similar to those of the previous closed study.

These data are from physicians and nurses across Japan and its various regions (from Hokkaido in the north to Okinawa in the south of Japan), and in examining past literature, few studies have focused on physicians and nurses evaluating the competencies of pharmacists worldwide. Many assessments have been conducted by peer evaluation of pharmacists or pharmacy universities [11,12,13]. Therefore, although these data are from only one country, we believe that this research is necessary to facilitate interprofessional collaboration and to reflect these results in pharmacy education by allowing physicians and nurses to assess the competencies of pharmacists and to understand their needs.

## 5. Conclusions

A global pandemic occurred, changing citizens’ awareness of public health and infectious diseases and significantly changing the healthcare system. With such substantive changes, pharmacy education must be reviewed based on the needs of not only citizens and regulators but also other healthcare professionals. The results of this study revealed that physicians and nurses have further expectations for pharmacists. We hope that these results will also be considered in regard to pharmacy education in other countries. The six-year Japanese pharmacy education curriculum, which is based on the model core curriculum, was first implemented in 2006 and then revised in 2015. Notably, a new model core curriculum will start in 2024. We expect that this study will provide useful information for the new 2024 model core curriculum in Japan.

## Figures and Tables

**Table 1:** Questionnaire Items.

Items 1–11: Pharmacists’ competencies ^1^
Item 1: Competency 1. Professionalism: To address the legal, ethical, and professional responsibilities of pharmacists.
Item 2: Competency 2. Patient-oriented attitude: To respect the rights of individuals and to promote the health and welfare of patients and consumers.
Item 3: Competency 3. Instruction to patients (Communication skills): To communicate effectively with patients, consumers, and other healthcare professionals to provide valuable information to them.
Item 4: Competency 4. Interprofessional collaboration: To collaborate with the healthcare teams in hospitals and regional communities
Item 5: Competency 5. Basic sciences: To understand the effects of medicines and chemicals on living bodies and environments. Competency 5–1: Fundamental basic science knowledge (more than a physician or nurse) Competency 5–2: Pharmaceutical knowledge (more than a physician or nurse)
Item 6: Competency 6. Medication therapy management: To contribute to the optimal use of medicines through pharmaceutical care. Competency 6–1: Prescription analytical skill or case analytical skill (more than a physician or nurse) Competency 6–2: Problem solving
Item 7: Competency 7. Community health and medical care: To contribute to public health and pharmaceutical hygiene and to enhance community healthcare and home care.
Item 8: Competency 8. Research competency: To engage in research on drug development and use of medicines to improve the healthcare environment.
Item 9: Competency 9. Lifelong learning: To enhance continuing professional development throughout their lifetime in response to advances in the healthcare environment.
Item 10: Competency 10. Education and training: To possess the will and attitude to advance the excellence of pharmacists of the next generation.
Item 11: Pharmacists have more competency than previously.
**Items 12–14: Needs of Physicians and Nurses**
Item 12: Pharmacists ready to work in medical field.
Item 13: You want to research with pharmacists.
Item 14: Pharmacists should play more of a role as medical professionals.
① Medication instruction and patient education
② Drug management and patient monitoring in clinical trials
③ Contribution to medical care as scientists (drug discovery, etc.)
④ Medical safety
⑤ Suggestions regarding prescriptions as a member of an interprofessional team
⑥ Basic and clinical research for medical development
⑦ Educational activities related to “health food products” or “health products” in the community
⑧ Activities on “disease prevention” or “public health” in the community
⑨ “Assessment and measurement of blood drug concentration” or “genetic diagnosis”
⑩ Medication instruction and drug management in home care

^1^ Some of the expressions for the professional competencies for pharmacists have been changed based on the English version of the revised model core curriculum for pharmacy education 2015 drafted on 15 November 2017 by Koichiro Ozawa, the Pharmaceutical Society of Japan.

**Table 2:** Characteristics of Respondents.

Characteristics of Respondents	Category	Number (%)	
		Physician (*n* = 304)	Nurse (*n* = 336)
Q1. Place of work	Clinic	101 (33.2%)	109 (32.4%)
	University hospital	86 (28.3%)	100 (29.8%)
	National public hospital	66 (21.7%)	59 (17.6%)
	General hospital (other than the above)	51 (16.8%)	68 (20.2%)
Q2. Relationship with pharmacists in daily work	Usually	18 (5.9%)	29 (8.6%)
	Often	80 (26.3%)	88 (26.2%)
	Sometimes	80 (26.3%)	83 (24.7%)
	Rarely	126 (41.4%)	136 (40.5%)

**Table 3:** Physicians’ and nurses’ assessments of competency in pharmacists.

Question Items	1 Agree Number (%)		2 Slightly Agree Number (%)		3 Slightly Disagree Number (%)		4 Disagree Number (%)		Mean (S.D.)		Mann–Whitney U Test
	P	N	P	N	P	N	P	N	P	N	
Competency 1: Professionalism	118 (38.8%)	118 (35.1%)	157 (51.6%)	161 (47.9%)	24 (7.9%)	55 (16.4%)	5 (1.6%)	2 (0.6%)	1.72 (0.04)	1.82 (0.04)	0.07
Competency 2: Patient-oriented attitude	106 (34.9%)	90 (26.8%)	149 (49.0%)	161 (47.9%)	44 (14.5%)	76 (22.6%)	5 (1.6%)	9 (2.7%)	1.83 (0.04)	2.01 (0.04)	0.00 ^※※^
Competency 3: Instruction to patients (communication skill)	117 (38.5%)	142 (42.3%)	165 (54.3%)	159 (47.3%)	19 (6.3%)	32 (9.5%)	3 (1.0%)	3 (0.9%)	1.70 (0.04)	1.69 (0.04)	0.70
Competency 4: Interprofessional collaboration	95 (31.3%)	104 (31.0%)	170 (55.9%)	163 (48.5%)	33 (10.9%)	64 (19.0%)	6 (2.0%)	5 (1.5%)	1.84 (0.04)	1.91 (0.04)	0.20
Competency 5–1: More fundamental basic science than P or N	30 (9.9%)	104 (31.0%)	83 (27.3%)	186 (55.4%)	165 (54.3%)	42 (12.5%)	26 (8.6%)	4 (1.2%)	2.62 (0.05)	1.84 (0.04)	0.00 ^※※^
Competency 5–2: More pharmaceutical knowledge than P or N	118 (38.8%)	240 (71.4%)	143 (47.0%)	82 (24.4%)	35 (11.5%)	13 (3.9%)	8 (2.6%)	1 (0.3%)	1.78 (0.04)	1.33 (0.03)	0.00 ^※※^
Competency 6–1: Prescription analytical skill or case analytical skill	15 (4.9%)	77 (22.9%)	118 (38.8%)	183 (54.5%)	145 (47.7%)	70 (20.8%)	26 (8.6%)	6 (1.8%)	2.60 (0.04)	2.01 (0.04)	0.00 ^※※^
Competency 6–2: Problem-solving	34 (11.2%)	55 (16.4%)	166 (54.6%)	161 (47.9%)	94 (30.9%)	109 (32.4%)	10 (3.3%)	11 (3.3%)	2.26 (0.04)	2.23 (0.04)	0.61
Competency 7: Community health and medical care	62 (20.4%)	78 (23.2%)	165 (54.3%)	151 (44.9%)	71 (23.4%)	98 (29.2%)	6 (2.0%)	9 (2.7%)	2.07 (0.04)	2.11 (0.04)	0.46
Competency 8: Research competency	38 (12.5%)	81 (24.1%)	150 (49.3%)	177 (52.7%)	107 (35.2%)	72 (21.4%)	9 (3.0%)	6 (1.8%)	2.29 (0.04)	2.01 (0.04)	0.00 ^※※^
Competency 9: Lifelong learning	71 (23.4%)	106 (31.5%)	187 (61.5%)	172 (51.2%)	38 (12.5%)	52 (15.5%)	8 (2.6%)	6 (1.8%)	1.94 (0.04)	1.88 (0.04)	0.18
Competency 10: Education and training	64 (21.1%)	81 (24.1%)	188 (61.8%)	174 (51.8%)	46 (15.1%)	74 (22.0%)	6 (2.0%)	7 (2.1%)	1.98 (0.04)	2.02 (0.04)	0.48
Pharmacists are ready to work in the medical field	107 (35.2%)	102 (30.4%)	154 (50.7%)	168 (50.0%)	37 (12.2%)	63 (18.8%)	6 (2.0%)	3 (0.9%)	1.81 (0.04)	1.90 (0.04)	0.07
Do you want to conduct research with pharmacists?	39 (12.8%)	45 (13.4%)	125 (41.1%)	132 (39.3%)	120 (39.5%)	128 (38.1%)	20 (6.6%)	31 (9.2%)	2.4 (0.05)	2.43 (0.05)	0.65
Pharmacists should play a greater role as medical professionals	71 (23.4%)	99 (29.5%)	170 (55.9%)	174 (51.8%)	60 (19.7%)	57 (17.0%)	3 (1.0%)	6 (1.8%)	1.98 (0.04)	1.91 (0.04)	0.14

P: Physicians, *n* = 304, N: Nurses, *n* = 336, ^※※^: *p* < 0.01.

**Table 4:** Principal component analysis (physician).

Question Items	Physician	
	Component 1	Component 2
Competency 9: Lifelong learning	0.821	−0.309
Competency 1: Professionalism	0.795	0.025
Competency 2: Patient-oriented attitude	0.789	0.032
Competency 4: Interprofessional collaboration	0.783	−0.181
Competency 10: Education and training	0.78	−0.312
Competency 7: Community health and medical care	0.767	−0.236
Competency 6–2: Problem solving	0.766	−0.002
Competency 8: Research competency	0.756	−0.145
Competency 3: Instruction to patients (communication skills)	0.73	−0.05
Competency 5–2: More pharmaceutical knowledge than P/N *	0.626	0.434
Competency 6–1: Prescription analytical skill or case analytical skill	0.597	0.519
Competency 5–1: More fundamental basic science than P/N *	0.567	0.602
Contribution ratio	54.17	9.38
Cumulative contribution ratio	54.17	63.55

*; P/N: Physicians, *n* = 304, N: Nurses, *n* = 336, Communality was set to 1, and a principal component loading of 0.50 was used as the cut-off point.

**Table 5:** Principal component analysis (Nurse).

Question Items	Nurse	
	Component 1	Component 2
Competency 9: Lifelong learning	0.868	−0.095
Competency 4: Interprofessional collaboration	0.820	−0.143
Competency 8: Research competency	0.814	−0.203
Competency 10: Education and training	0.812	−0.156
Competency 7: Community health and medical care	0.811	−0.238
Competency 6–2: Problem solving	0.790	−0.129
Competency 1: Professionalism	0.786	−0.025
Competency 2: Patient-oriented attitude	0.773	−0.153
Competency 3: Instruction to patients (communication skill)	0.742	0.005
Competency 5–2: More pharmaceutical knowledge than P/N *	0.708	0.325
Competency 5–1: More fundamental basic science than P/N *	0.626	0.588
Competency 6–1: Prescription analytical skill or case analytical skill	0.530	0.607
Contribution ratio	58.05	8.434
Cumulative contribution ratio	58.05	66.49

*; P/N: Physicians, *n* = 304, N: Nurses, *n* = 336, Communality was set to 1, and a principal component loading of 0.50 was used as the cut-off point.

**Table 6:** Pharmacists should play an even greater role.

Question Items	1. Should Work Harder and Play a Greater Role		2. Should Play a Greater Role		3. Play Their Role		4. Sufficiently Play Their Role		Mean (S.D.)		Mann–Whitney U Test
	P	N	P	N	P	N	P	N	P	N	
1 Medication instruction and education for patients	69 (22.7%)	84 (25.0%)	82 (27.0%)	128 (38.0%)	130 (42.8%)	96 (28.6%)	23 (7.6%)	28 (8.3%)	2.35 (0.91)	2.20 (0.91)	0.02
2 Genetic diagnosis/Measurement and evaluation of blood drug concentration	50 (16.4%)	57 (17.0%)	117 (38.5%)	124 (36.9%)	115 (37.8%)	127 (37.8%)	22 (7.2%)	28 (8.3%)	2.36 (0.84)	2.38 (0.86)	0.81
3 Disease prevention/public health (activities) in the community	54 (17.8%)	76 (22.6%)	130 (42.8%)	145 (43.2%)	104 (34.2%)	101 (30.1%)	16 (5.3%)	14 (4.2%)	2.27 (0.81)	2.16 (0.82)	0.08
4 Pharmaceutical awareness activities for citizens (including health foods)	47 (15.5%)	69 (20.5%)	129 (42.4%)	145 (43.2%)	108 (35.5%)	98 (29.2%)	20 (6.6%)	24 (7.1%)	2.33 (0.82)	2.23 (0.86)	0.09
5 Basic/clinical research for the development of medical care	52 (17.1%)	70 (20.8%)	119 (39.1%)	122 (36.3%)	115 (37.8%)	114 (33.9%)	18 (5.9%)	30 (8.9%)	2.33 (0.83)	2.31 (0.90)	0.73
6 Prescription proposal for treatment (as a member of team medical care)	45 (14.8%)	72 (21.4%)	98 (32.2%)	118 (35.1%)	133 (43.8%)	116 (34.5%)	28 (9.2%)	30 (8.9%)	2.47 (0.86)	2.31 (0.91)	0.02 ^※^
7 Medical safety	36 (11.8%)	71 (21.1%)	100 (32.9%)	116 (34.5%)	136 (44.7%)	109 (32.4%)	32 (10.5%)	40 (11.9%)	2.54 (0.84)	2.35 (0.94)	0.01 ^※^
8 Medical contribution in drug discovery/as a scientist	55 (18.1%)	63 (18.8%)	103 (33.9%)	116 (34.5%)	124 (40.8%)	130 (38.7%)	22 (7.2%)	27 (8.0%)	2.37 (0.86)	2.36 (0.88)	0.83
9 Drug management and patient monitoring related to clinical trials, etc.	41 (13.5%)	66 (19.6%)	102 (33.6%)	107 (31.8%)	132 (43.4%)	130 (38.7%)	29 (9.5%)	33 (9.8%)	2.49 (0.84)	2.39 (0.91)	0.15
10 Drug management and medication instruction in home medical care	49 (16.1%)	90 (26.8%)	96 (31.6%)	110 (32.7%)	129 (42.4%)	105 (31.3%)	30 (9.9%)	31 (9.2%)	2.46 (0.88)	2.23 (0.95)	0.00 ^※※^

Physician (P): *n* = 304, Nurse (N): *n* = 336, ^※※^: *p* < 0.01, ^※^: *p* < 0.05.

## Data Availability

Not applicable.
